# Clinicopathological Features of Meningioangiomatosis Associated with Meningioma: A Case Report with Literature Review

**DOI:** 10.1155/2012/296286

**Published:** 2012-11-05

**Authors:** Huajuan Cui, Huijuan Shi, Xiaodong Chen, Wei Wang, Riquan Lai, Anjia Han

**Affiliations:** ^1^Department of Pathology, Guangzhou General Hospital of PLA, Guangzhou 510010, China; ^2^Department of Pathology, The First Affiliated Hospital and Zhongshan School of Medicine, Sun Yat-sen University, Guangzhou 510080, China

## Abstract

*Aim*. To analyze the clinicopathological features of meningioangiomatosis (MA) associated with meningioma. *Methods*. We present one case of MA associated with meningioma. Histopathological examination and immunohistochemistry were used. *Results*. The age of the patient was 33-year-old man. Histopathologically, MA was characterized by vascular proliferation with perivascular meningothelial cells and/or fibroblast proliferation, entrapped glial islands. In addition, MA was associated with transitional meningioma. The patient was alive without evidence of recurrence at 18 months after mass resection. *Conclusion*. MA associated with meningioma is an extremely rare lesion. The differential diagnosis includes cortical invasion by meningioma and intracerebral schwannoma. Patients with MA associated with meningioma often have a good prognosis after operation.

## 1. Introduction

Meningioangiomatosis (MA) is characterized by a vascular proliferation with perivascular growths of cells of uncertain histologic origin but variously hypothesized to be of either meningothelial or fibroblastic lineages along with entrapped neuroglial islands and focal calcifications. MA is a rare meningovascular harmartomatous lesion. It may occur sporadically or occur in association with neurofibromatosis type 2 (NF2) [1, 2]. MA is characterized by a plaque-like or nodular mass within the cerebral cortex and overlying leptomeninges in patients with intractable seizure and headache. MA associated with meningioma is extremely rare. To our knowledge, 30 cases of MA associated with meningioma have been reported in the English literature. We present one case of MA with meningioma and analyzed its clinicopathological features with a brief literature review.

## 2. Case Presentation

### 2.1. Clinical Evaluation

After reviewing the surgical pathology database from Department of Pathology, Guangzhou General Hospital of PLA, Guangzhou, China, we found one case of MA associated with meningioma. The data on clinical features, imaging evidence including computerized tomography (CT) and magnetic resonance imaging (MRI), and treatment were collected. The follow-up information was available for the patient.

### 2.2. Histopathologic Examination and Immunohistochemistry

Tissue for light microscopy was fixed in 10% neutral buffered formalin and embedded in paraffin by use of routine procedures. Four-micrometer-thick sections were cut from the tissue blocks and stained with hematoxylin-eosin.

Immunohistochemistry staining was carried out on formalin-fixed, paraffin-embedded tissue using EnVison Kit (Dako, Carpinteria, CA, USA). The following primary antibodies (Dako, Carpinteria, CA, USA) were used: epithelial membrane antigen (clone: E29), vimentin (clone: v9), CD34 (clone: QBEnd 10), S-100 protein, neuron-specific enolase (clone: BBS/NC/VI-H14), neurofilament (clone: DA2), cytokeratin (clone: AE1/AE3), cytokeratin 19 (clone: RCK108), CD99 (clone: 12E7), progesterone receptor (clone: PgR 636 ), Bcl-2 (clone: 124), glial fibrillary acidic protein (clone: 6F2), and Ki-67 (clone: MIB1). Appropriate positive and negative control (phosphate buffered solution was substituted for the primary antibody) slides were employed.

### 2.3. Literature Review

All English papers about meningioangiomatosis with meningioma published on PubMed were reviewed, and the surgical pathology information on sex, age, location of lesion, clinical presentation, status of neurofibromatosis type 2, histopathological type of meningioma, and clinical outcome were obtained.

## 3. Results

### 3.1. Clinical Features

**Figure 1:**
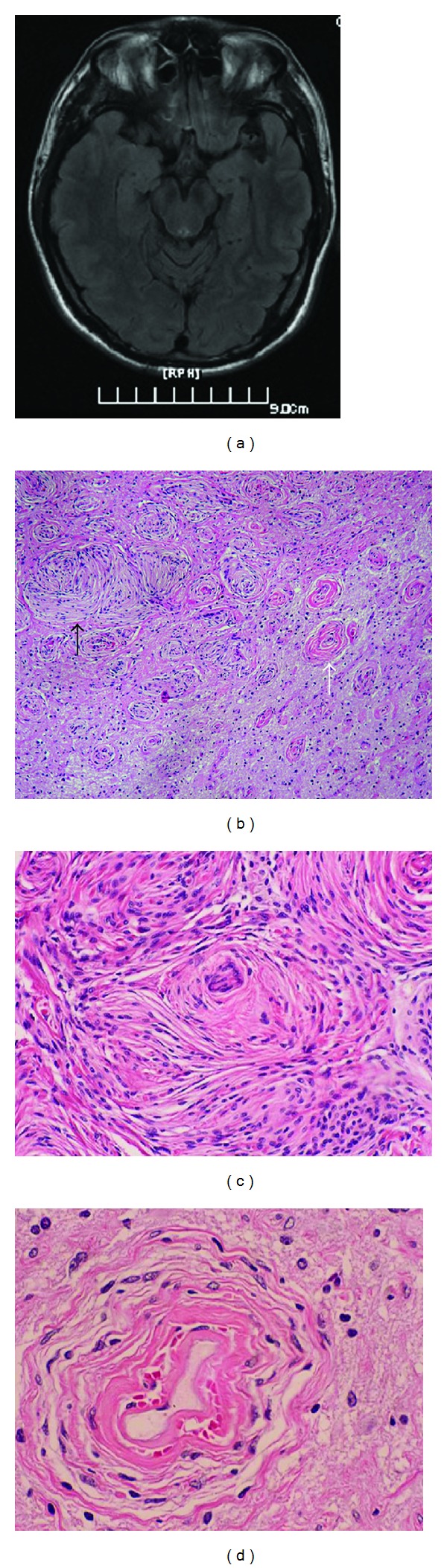
(a) MRI shows a low-signal intensity in the left temporal lobe on T1-weighted image; (b) transition area between MA (white arrow) and meningioma (black arrow), HE ×40; (c) transitional meningioma, HE ×200; (d) MA area is typical of collagenized vessels cuffed by meningothelial cells or fibroblasts under high power (HE ×400).

A 33-year-old man was admitted to our emergency room for hyperspasmia of extremities without consciousness suddenly. He had felt weary for one year. CT indicated calcification in left temporal lobe. MRI showed a mass measuring 20 × 15 mm in left temporal lobe and indicating vascular malformation. He had no family history or stigmata of neurofibromatosis. The symptoms disappeared when carbamazepine was used for two months. After treatment, the patient had hyperspasmia of extremities without consciousness for five minutes again three months ago. The patients had four times seizures which persisted 5 to 15 minutes each time in the past three months. MRI showed a slight low signal intensity on T1-weighted image and low-signal intensity on T2-weighted image (Figure 1). The patient underwent mass resection. The mass, measuring 20 × 15 × 5 mm in size, was firm and gray with abundant blood vessels. The patient remained free of disease and no evidence of tumor recurrence at 18 months after surgery.

### 3.2. Histopathologic Findings and Immunohistochemistry Staining

The removed mass was composed of mainly meningothelial cells forming small whorls intermixed with spindle cells. Psammoma bodies were found in the center of whorls. Neither necrosis nor mitotic activity was found. The histopathological findings were consistent with transitional meningioma. The adjacent cerebral parenchyma showed prominent blood vessels including capillaries and middle-sized venules which were cuffed by meningothelial cells or fibroblasts with an onion-peel appearance. Degenerative neuron and gliosis were observed in the intervening brain parenchyma. The transition between meningioma and MA was not found (Figure 1).

Immunohistochemistry staining showed that tumor cells of meningioma were strongly immunoreactive for vimentin, epithelial membrane antigen, and progesterone receptor. The perivascular meningothelial cells or fibroblasts were negative for epithelial membrane antigen and progesterone receptor. MIB-1 index was approximately 1% in MA area, but 3% in meningioma area. Glial fibrillary acidic protein, CD99, CD34, S-100 protein, neuron specific enolase, neurofilament, cytokeratin, cytokeratin 19, and Bcl-2 were negative in MA and meningioma area.

## 4. Discussion

MA is a rare benign entity and was first described by Bassoe and Nuzum in 1915 as an incidental autopsy finding in a 15-year-old boy with NF2 [3]. Although MA is originally described in association with NF2, sporadic MA occurs more common than that associates with NF2 [2]. Sporadic MA is usually a single lesion with long history of seizures. NF2-associated MA may occur in multiple lesions and is usually asymptomatic. 

Rarely, MA has been described to coexist with meningioma [4–13], vascular malformations [14], encephalocele [15], oligodendroglioma [16], and meningeal haemangiopericytomas [9]. Among these, meningiomatosis with meningioma is the most frequent combination. Up to date, we found 31 cases of MA associated with meningioma including our current case in the English literature [4–13, 17–21] (Table 1). The ratio of male to female was 4.2 : 1. MA associated with meningioma occurred frequently in young patients with the mean age of 15 years ranging from 9 months to 58 years and the median age was 17 years old. The most common symptoms of patients with MA associated with meningioma were seizures and headache. Frontal or temporal cortex was most often involved. Histopathologically, the meningiomas were mostly of the transitional subtype (13 cases), though there were 6 fibroblastic, 4 meningothelial, 5 atypical, 1 microcystic, and 1 sclerosing variant.

The radiographic findings of MA may have a variety of features. The common findings on CT scan are iso- to slightly hyperintense that show variable amount of calcification with little or no contrast enhancement. MRI reveals iso or hypointensity on T1-weighted image and heterogeneous cortical mass surrounded by an area of increased intensity on T2-weighted images, probably due to edema or gliosis. The presence of calcification on CT and a low-signal intensity rim on T2-weighted MRI images are the most helpful features that suggest the diagnosis of MA [9, 22].

The differential diagnosis of MA associated with meningioma includes cortical invasion by meningioma and intracerebral schwannoma [9, 23]. Cortical invasion by meningioma occurring along the vessels in Virchow-Robin spaces does not technically represent parenchymal invasion but may indicate an increased probability of recurrence. True invasion is present when tumor cells break through the pia mater to involve underlying cerebral cortex and encircle islands of heavily gliotic tissue. Moreover, invasive meningioma usually has a serrated irregular outer border intermixed with cerebral parenchyma. MIB-1 index is lower in MA area than that in meningioma area, indicating that MA is not the invasion component of meningioma. Intracerebral schwannoma is very rare and has a distinctive plexiform growth pattern, and small aggregates of schwann cells spread extensively into the surrounding brain tissue along perivascular spaces adjacent to the tumor nodule [23]. Two kinds of lesions can be easily discriminated by immunohistochemistry staining. 

The pathogenesis of MA remains unclear. A developmental, dysplastic, hamartomatous, or reactive etiology is favored based on its typically benign clinical course and lack of significant proliferative activity in most cases. However, recent studies show that loss of 22q12 (NF2 gene) and loss of heterozygosity have been found in pure MA and MA associated with meningioma, suggesting that MA may be neoplastic in nature [10, 24, 25]. Perry et al. suggest that in most MA associated with meningioma, the MA component is neoplastic, likely representing an exuberant perivascular pattern of spread from the meningioma, rather than an underlying hamartoma. This pattern of spread may be facilitated by meningiomas that are predominantly leptomeningeal or intracerebral in origin [6]. As for this question, it needs further study. 

Surgery resection is an adequate therapy strategy for MA associated with meningioma. Adjuvant radiation therapy is recommended if incomplete surgical excision has been performed [26]. The prognosis of patients with MA associated with meningioma is associated with histopathological grade of meningioma and operation. Of 16 cases of MA associated with meningioma with follow-up information, 14 patients remained alive and no evidence of tumor recurrence. Only one patient died due to operative complication [19], and another one with a high MIB-index (up to 10%) and moderately increased cellularity had recurrence after surgery [9].

## Figures and Tables

**Table 1:** Previous reported and present case of MA associated with meningioma in the English literature.

Author (year)	Sex (M/F)	Age (years)	Location	Clinical presentation	NF2	Histological type of meningioma	Clinical outcome
Auer et al. (1982) [19]	M	15	Frontal lobe	Subarachnoid haemorrhage	—	Fibroblastic	Dead due to operative complication
Louw et al. (1990) [18]	M	15	Frontal lobe	Subarachnoid haemorrhage	—	Fibroblastic	NA
	M	33	Frontal lobe	Headache	—	Transitional	NA
Wilson et al. (1991) [17]	M	17	Frontal lobe	Headache and seizure	—	Transitional	NA
Blumenthal et al. (1993) [13]	M	0.9	Frontal lobe	Seizure	—	Transitional	No recurrence
Giangaspero et al. (1999) [12]	M	9	Temporal lobe	Asymptomatic	—	Transitional	No recurrence
	M	28	Frontal lobe	Seizure	—	Transitional	No recurrence and alive
Mut et al. (2000) [11]	F	20	Temporal lobe	Seizure	—	Transitional	No recurrence and alive
Sinkre et al. (2001) [10]	M	8	Frontal lobe	Headache	—	Atypical	No recurrence and alive
	M	3	Frontoparietal lobe	Seizure	—	Fibroblastic	No recurrence and alive
	M	4	Frontal lobe	Sudden headache	—	Fibroblastic	No recurrence and alive
Kim et al. (2002) [9]	M	6	Temporal lobe	Headache and facial palsy hemiparesis	—	Transitional	Recurrence and alive
	M	9	Frontal lobe	Seizure	—	Meningothelial	No recurrence and alive
	M	19	Temporal lobe	Seizure	—	Sclerosing	No recurrence and alive
Iezza et al. (2003) [8]	M	33	Frontal lobe	Seizure	—	Transitional	NA
Kuchelmeister et al. (2003) [7]	M	58	Frontal lobe	Headache and forgetfulness	—	Microcystic	No recurrence
	M	28	Temporal lobe	NA	—	Transitional	NA
	M	17	Frontal lobe	NA	—	Transitional	NA
	F	20	Temporal lobe	NA	—	Meningothelial	NA
	M	9 Mo.	NS	NA	—	Atypical	NA
Perry et al. (2005) [6]	M	16	NS	NA	—	Atypical	NA
	M	35	NS	NA	—	Transitional	NA
	M	23	NS	NA	—	Transitional	NA
	F	14	Temporal lobe	NA	—	Transitional	NA
	M	16 Mo.	Frontal lobe	NA	—	Atypical	NA
	M	7	Frontal lobe	NA	—	Transitional	NA
Deb et al. (2006) [5]	F	1.5	Temporal lobe	Seizure	—	Transitional	No recurrence
Saad et al. (2009) [4]	F	3	Frontal lobe	Seizure	—	NA	NA
Shi et al. (2011) [20]	F	50	Left temporal lobe	Headache and dizziness	—	Meningothelial	No recurrence
Chen et al. (2010) [21]	M	34	Right frontoparietal lobe	Progressive numbness and weakness of lower extremity	—	Atypical	No recurrence
Present	M	33	Temporal lobe	seizure	—	transitional	No recurrence and alive

MA: meningioangiomatosis; NA: not available; NF2: neurofibromatosis type 2; NS: cerebral, not specified.
